# Chemical Characterization and Anti-Inflammatory Activity of Phytoconstituents from *Swertia alata*

**DOI:** 10.3390/plants10061109

**Published:** 2021-05-31

**Authors:** Sakshi Bajaj, Shivkanya Fuloria, Vetriselvan Subramaniyan, Dhanalekshmi Unnikrishnan Meenakshi, Sharad Wakode, Avneet Kaur, Himangini Bansal, Satish Manchanda, Sachin Kumar, Neeraj Kumar Fuloria

**Affiliations:** 1 Delhi Institute of Pharmaceutical Science and Research, Pushp Vihar, New Delhi 110017, India; sakshibajaj84@gmail.com (S.B.); sharadwakode@gmail.com (S.W.); himanginibansal@gmail.com (H.B.); manchandasatish@gmail.com (S.M.); s1378n@gmail.com (S.K.); 2 Faculty of Pharmacy, AIMST University, Kedah 08100, Malaysia; 3 Faculty of Medicine, Bioscience and Nursing, MAHSA University, Kuala Lumpur 42610, Malaysia; drvetriselvan@mahsa.edu.my; 4 College of Pharmacy, National University of Science and Technology, Muscat 130, Oman; dhanalekshmi@nu.edu.om; 5 SGT College of Pharmacy, SGT University, Budhera, Gurugram 122505, India; avneetkaur1986@gmail.com

**Keywords:** *Swertia alata*, partitioned, chromatography, spectrometry, anti-inflammatory, ulcerogenic

## Abstract

*Swertia alata* C.B Clarke (Gentianaceae) is a well-reported plant in the traditional system of medicine. The present study was intended to isolate the phytoconstituents from the ethanolic extract of the aerial parts of *S. alata*; and evaluate for in vitro COX-1/COX-2 inhibition activity, in vivo anti-inflammatory and ulcerogenic activity. Phytoisolation involved partitioning of *S. alata* ethanolic extract into petroleum ether and chloroform soluble fractions using silica gel-based column chromatography. The isolation afforded two phytoisolates, namely oleanolic acid (SA-1) and 3-hydroxylup-12-(13)-ene-17-carboxylic acid (SA-4). Phytoisolates structures were established by melting point, ultraviolet (UV), attenuated total reflection-Fourier-transform infrared (ATR-FTIR), nuclear magnetic resonance (1H-NMR, 13C-NMR and HMBC) and mass spectrometry. Phytoisolates were further evaluated for in vitro cyclooxygenase (COX-1/COX-2) inhibitory activity, in vivo anti-inflammatory and ulcerogenic activity. The study revealed SA-4 (COX-1/COX-2 inhibition activity of 104/61.68 µM with % inhibition of 61.36) to be more effective than SA-1 (COX-1/COX-2 inhibition activity of 128.4/87.25 µM, with % inhibition of 47.72). SA-1 and SA-4, when subjected to ulcerogenic study, exhibited significant gastric tolerance. The current study reports chromatographic isolation and spectrometric characterization of SA-1 and SA-4. The present study concludes that compound SA-4 possess significant anti-inflammatory activity and less irritant property over gastric mucosa with no significant ulcerogenicity in comparison to indomethacin.

## 1. Introduction

*Swertia alata* C.B Clarke (Gentianaceae) is a perennial herb and widely distributed plant in the west and north-west Himalayas, particularly in Kashmir to Kumaon, Mussorie, Dehradun, and the Nainital region of India [1]. Numerous species of Swertia are being used as substituents or adulterants of *S. chirata*. *S. alata* is used and unknowingly collected as *S. chirata* by traders for the preparation of several ayurvedic drugs [2]. *S. alata* is widely used in indigenous medicine as it possesses various properties. The bitterness, anthelmintic, hypoglycemic, and antipyretic properties are attributed to amarogentin, swerchirin, swertiamarin, and other active principles of the herb [3]. The most potential specie of Swertia genus is *S. chirata,* which is now nearly extinct from India. *S. alata* is known to possess oleanolic acid [4], swertisin [4], swertiamarin [5], swertianin, [6] methyl swertianin [6], methylbellidifolin [6], bellidifollin [7] swertiaperennine, and decussatin [8] but still very limited data is available over of the phytochemistry of *S. alata.* Non-steroidal anti-inflammatory drugs (NSAIDs) are among the most widely utilized medications in the world due to their efficacy in reducing pain and inflammation [9]. Inflammation is a typical defensive response to tissue injury, which includes a complex array of enzyme activation, mediator release, fluid extravasations, cell migration, tissue breakdown, and repair [10]. Inflammatory injuries actuate the release of a variety of systemic mediators, cytokines, and chemokines, which increases the rate of synthesis of prostaglandin [11]. Its production relies upon the activity of prostaglandin G/H synthases, colloquially known as COXs, bifunctional enzymes that contain both cyclooxygenase and peroxidase activity, which exist in two distinct isoforms, namely COX-1 and COX-2 [12]. The essential method of activity is inhibition of the pro-inflammatory enzyme cyclooxygenase (COX). NSAIDs as a class also involves the traditional nonselective NSAIDs (tNSAIDs) that nonspecifically repress both COX-1 and COX-2, and selectively inhibits COX-2. Albeit effective at relieving pain and inflammation, tNSAIDs are related with a significant risk of serious gastrointestinal adverse effects [13]. To overcome these adverse effects, specific inhibitors of the COX-2 isoenzyme were created, which opens the possibility to provide anti-inflammatory and analgesic advantages while hypothetically leaving the gastro protective activity of the COX-1 isoenzyme in place. However, vital concerns have recently been raised with respect to the potential cardiovascular toxicity of COX-2 inhibitors [14]. Looking for selective COX-2 inhibitors without affecting the normal physiological functions of COX-1 has remained a noteworthy thrust area of anti-inflammatory pharmaceutical research. Nevertheless, the anti-inflammatory agents with greater activity towards COX-2 yet less receptive activity towards COX-1 are acknowledged as novel anti-inflammatory agents in the mainstream of anti-inflammatory research [15]. Due to the inherent problems associated with the current non- steroidal as well as steroidal anti-inflammatory agents, there is a continuous search for phytoconstituents having anti-inflammatory activity with reduced gastrointestinal side effects. Evidence suggests that in the past, Bajaj et al. isolated nonacosyl triacontanoate and 8-O-b-D-glucopyranosyl-(2-acetyl)-1,3-dihydroxy-5-methoxy-xanthone in *S. alata* plant and evaluated for in vitro COX-1/2, in vivo anti-inflammatory and ulcerogenic activity. The study revealed the significant anti-inflammatory potential of the isolates [16]. Based on the afore mentioned investigations, the present study was designed to further explore the presence of any new phytoisolate in the aerial parts of *S. alata* plant as a potential anti-inflammatory agent.

## 2. Results

The partitioning of *S. alata* ethanolic extract into petroleum ether and chloroform soluble fractions using silica gel-based column chromatography offered two phytoisolates: SA-1 and SA-4. The structural elucidations of isolated compounds were achieved by melting point, ultraviolet (UV), attenuated total reflection-Fourier-transform infrared (ATR-FTIR), nuclear magnetic resonance (1H-NMR, 13C-NMR and HMBC), and mass spectrometry.

### 2.1. Structure Elucidation of Compounds

#### 2.1.1. Oleanolic Acid (SA-1)

**Figure 1:**
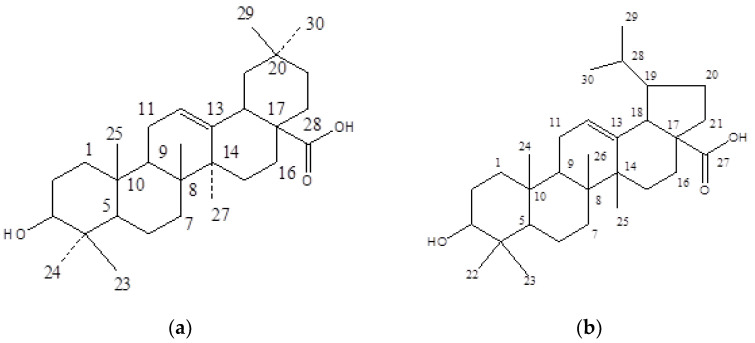
The chemical structures of (**a**) SA-1; and (**b**) SA-4.

The phyotoisolate SA-1 (Figure 1a) was obtained as white amorphous powder from elution of the column with chloroform: petroleum ether (2:8), 50 mg (0.016%), and having a melting point of 71 °C. 

**Figure 2:**
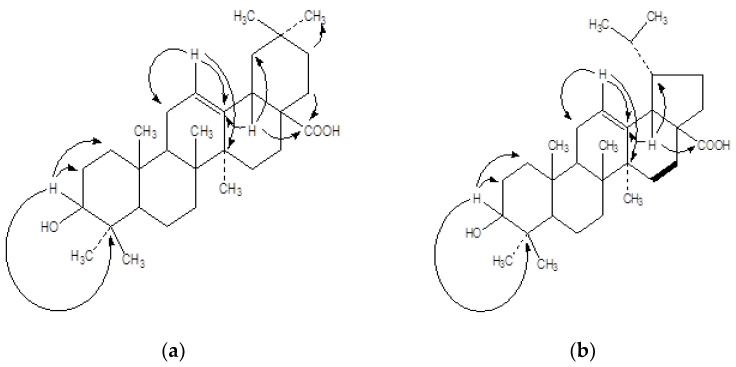
HMBC interaction in (**a**) SA-1 (**b**) SA-4.

The SA-1 exhibited ATR-FTIR (cm^−1^) bands at 2917, 2849, 1730, 726; ^1^H NMR and ^13^C NMR signals (data given in Table 1); ESI-MS signals at *m*/*z*: 457 [M+H]^+^ and 456 [M]^+^; and HMBC interaction (Figure 2a).

#### 2.1.2. 3-Hydroxylup-12-(13)-ene-17-carboxylic Acid (SA-4)

The phyotoisolate SA-4 (Figure 1b) was obtained as yellow amorphous powder from elution of the column with chloroform: petroleum ether (3:7), 86 mg (0.071%), and having a melting point of 110 °C. The SA-4 exhibited ATR-FTIR (cm^−1^) bands at 2917, 2849, 1730, 724; ^1^H NMR and ^13^C NMR signals (data given in Table 2); ESI-MS signals at *m*/*z* 456 [M]^+^ and 455 [M-H]^+^); and HMBC interaction (Figure 2b). 

### 2.2. Biological Activity

#### 2.2.1. In Vitro COX-1 and COX-2 Inhibitory Assay 

The inhibitory activity of isolated compounds (SA-1 and SA-4) was evaluated against ovine COX-1 and human recombinant COX-2 (using enzyme immunoassay kit) to determine IC_50_ (µM) values (Table 3). 

#### 2.2.2. In Vivo Anti-Inflammatory Activity

The degree of swelling of the carrageenan injected paws was maximal at the third hour after injection. Statistical analysis revealed that SA-4 (8 mg/kg) significantly inhibited the development of edema at the third hour after treatment (*p* < 0.05). The biological data for SA-1 and SA-4 is shown in Table 4, which clearly implies that the isolated compounds exhibited varying degree of anti-inflammatory activity.

#### 2.2.3. Ulcerogenic Activity

**Figure 3:**
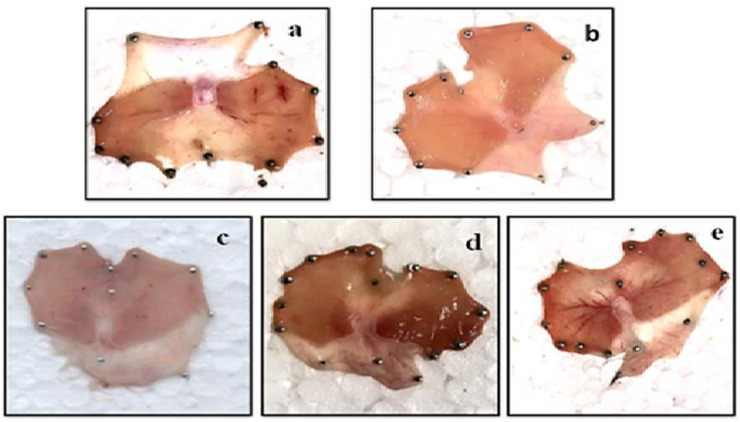
Macroscopic observation of gastric mucosal lesions in the ulcer model; (**a**) Indomethacin (21 mg/kg); (**b**) control; (**c**) SA-4 (12 mg/Kg); (**d**) SA-4 (24 mg/kg); (**e**) SA-1 (24 mg/Kg).

The results presented in Table 5 reveals compound SA-1 and SA-4 to possess a better gastrointestinal safety profile with an ulcer score of 2.5 ± 0.61 and 1.8 ± 0.44, respectively, in comparison to the standard drug Indomethacin 2.7 ± 0.273. Further, SA-4 possesses significantly less ulcerogenic potential as compared with SA- 1 in a dose of 12 mg/kg. The macroscopic view to support ulcerogenic activity of compound SA-1 and SA-4 is also presented in the Figure 3.

## 3. Discussion

### 3.1. Isolation of Phytoconstituents

**Figure 4:**
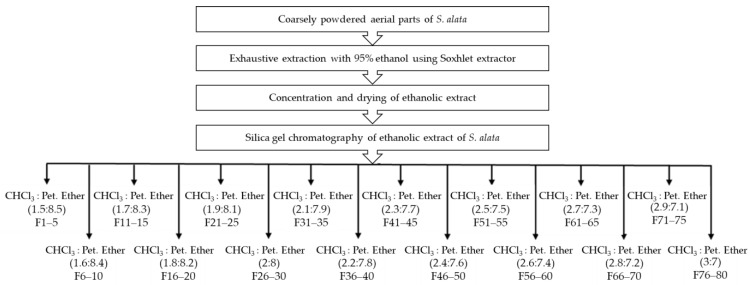
Flowchart representing the extraction and isolation of phytoisolates from *S. alata* extract using various fractions of chloroform and petoleum ether as eluent.

The current study intended to explore any new phytoisolate present in the aerial parts of *S. alata* plant. For this purpose, 50 g of prepared extract of *S. alata* was successively eluted with chloroform and petroleum ether using normal phase column chromatography. This yielded 16 fractions (each fraction of 400 mL) using eluent system of chloroform: petroleum ether in different ratio in various fractions, such as: F1–5 (1.5:8.5), F6–10 (1.6:8.4), F11–15 (1.7:8.3), F16–20 (1.8:8.2), F21–25 (1.9:8.1), F26–30 (2:8), F31–35 (2.1:7.9), F36–40 (2.2:7.8), F41–45 (2.3:7.7), F46–50 (2.4:7.6), F51–55 (2.5:7.5), F56–60 (2.6:7.4), F61–65 (2.7:7.3), F66-70 (2.8:7.2), F71-75 (2.9:7.1), and F76-80 (2:8). The fractions 26–30, when combined and exposed to TLC, offered a new spot with Rf value of 0.49 (chloroform:methanol, 3:2). The fraction 26–30, when dried and subjected to purification by preparative TLC, offered pure white amorphus mass SA-1. Whereas, the fractions 76–80 when combined and exposed to TLC, offered a new spot with Rf value of 0.53 (chloroform:methanol, 4:1). The fraction 76–80, when dried and subjected to purification by preparative TLC, offered pure yellow amorphus mass SA-4. The flow chart given in Figure 4, presents the extraction and isolation of phytoisolates from *S. alata* plant using various fractions. 

### 3.2. Characterization of Phyto-Isolates

The compound SA-1, identified as oleanolic acid, was obtained as white amorphous powder. It was acid hydrolysed and responded positively to the Liebermann-Burchard test for steroids. The presence of sterol nucleus was confirmed by acid hydrolysis using 2M H_2_SO_4_. The IR spectrum showed the characteristic absorption bands at (2917, 2849) for C-H stretching, and (1730) for C=O stretching and thereby confirmed the presence of the carbonyl group. The generation of the prominent ion peaks at *m*/*z* 457 [C_30_H_48_O_3_]^+^ was attributed to the molecular ion of oleanolic acid. Other fragment ions peaks at *m*/*z* 439 [C_30_H_47_O_2_]^+^, 411 [C_29_H_47_O]^+^, 395 [C_29_H_47_]^+^, 248 [C_16_H_24_O_2_]^+^, 235 [C_15_H_24_O_2_]^+^ supported the presence of steroidal structure.

The ^1^H NMR spectrum of compound SA-1, methyl protons displayed one singlet at δ 0.47 for 6H assigned to H-29 and H-30 protons, two singlets at δ 0.4 and 0.45 assigned to H-23 and H-24 protons, and three singlets at δ 0.52, 0.78, 0.81 were assigned to H25, H-26, and H-27 protons, respectively. There was a characteristic triplet signal at δ 5.21 with *J* value of 15.12 assigned to H-12. The protons of OH and COOH group displayed a characteristic broad signal at δ 12.42 and 12.6, respectively. The remaining methylene and methine unit protons displayed broad multiplet signals between δ0.47 and 2.13. The ^13^C NMR spectrum data of SA-1 exhibited important signals at δ 14.21 for C-23 and C- 24, signal at δ 24.71 for C-29 and C-30. The signal at δ 29.06 was assigned to C-26 and C-27. A signal at δ 22.71 was assigned to C-25, the signal at δ 32.02 was assigned to C-31, and the signal at 178 was assigned to carbonyl carbon. The two characteristic signals at δ 125.77 and 127.28 were assigned to C12 and C13, respectively. The carbonyl group signal appeared at δ 178.45. The remaining 19 carbons displayed their signals from δ 14.1 to 77.02 (Table 1). 

The HMBC correlations of H-C(3) with C(1), C(2), C(4), C(23), and C(24), of H-C(9) with C(5), C(8), and C(10), of H-C(12) with C(11), C(13), C(14), and C(18), of H-C(18) with C(13), C(14), C(16), and C(28), and of H-C(22) with C(28), indicated the relative positions of these groups in the molecule SA-1 (Figure 2a). 

The IR data, mass fragmentation pattern, ^1^H NMR, and ^13^C NMR chemical shifts of the isolated compound SA-1 were comparable with related compound viz, oleanolic acid. Based on spectral data analysis and acid hydrolysis based chemical identification, the structure of isolated compound SA-1 was finally elucidated as oleanolic acid.

The compound SA-4 identified as 3-hydroxylup-12-(13)-ene-17-carboxylic acid, was obtained as yellow amorphous powder. It was obtained chemically by acid hydrolysis. It responded positively to the Liebermann-Burchard test for steroids and formed effervescence with sodium bicarbonate solution, indicating the presence of carboxylic acid group in the molecule. The chemical identification was done by acid hydrolysis method using 2M H_2_SO_4,_ which confirmed the presence of sterol nucleus. The IR spectrum showed characteristic absorption bands for (2917, 2848) for C-H stretching, and (1735) for C=O stretch to confirm the presence of carbonyl group. The +ve ion mass spectrum showed a molecular ion peak at *m*/*z* 456 [C_30_H_48_O_3_]^+^ corresponding to M^+^ ion, and 455 [C_30_H_47_O_3_]^+^ corresponds to M-1 peak, which indicated saturated nature of molecule. The characteristic signals at *m*/*z* 439 [C_30_H_47_O_2_]^+^, 411 [C_29_H_47_O]^+^, 395 [C_29_H_47_]^+^, and 235 [C_15_H_24_O_2_]^+^ suggested the presence of steroidal structure.

The ^1^H-NMR spectrum of compound SA-4, methyl protons displayed one singlet at δ 0.65 for 6H assigned to H-29 and H-30, two singlets at δ 0.81 and 0.85 assigned to H-22 and H-23 protons, respectively, and one singlet at δ 0.95, which was assigned to H24, H-25, and H-26 protons. There was a characteristic triplet signal at δ 5.20 with *J* value of 15.1 Hz assigned to H-12. The protons of hydroxy and COOH group displayed characteristic broad signal at δ 12.49 and 12.56, respectively. The remaining methylene and methine unit protons displayed broad multiplet signals at δ 0.95-3.72. The ^13^C NMR spectrum data of SA-4 exhibited important signals for two methyl carbons at δ 17.97 C-22, C-23, C-29, and C-30. The signal at δ 27.2 was assigned to C-26 and C-27. A signal at 18.01 was assigned to C-25. The signal δ 178 was assigned to carbonyl carbon. The two characteristic signals at δ 125.42 and 127.35 were assigned to C12 and C13, respectively. The remaining 19 carbons displayed their signals from δ 14.1 to 72.53 (Table 2). 

The HMBC correlations of H-C(3) with C(1), C(2), C(4), C(23), and C(24), of H-C(9) with C(5), C(8), and C(10), of H-C(12) with C(11), C(13), C(14), and C(18), of H-C(18) with C(13), C(14), C(16), C(19) and C(27), and of H-C(21) with C(27), indicated the relative positions of these groups in the molecule SA-4 (Figure 2b). 

The IR, data, mass fragmentation pattern, ^1^H and ^13^C NMR chemical shifts of the isolated compound SA-4 were comparable with related compound viz and hydroxylupic acid. Based on spectral data analysis and acid hydrolysis-based chemical identification, the structure of isolated compound SA-4, was finally elucidated as 3-hydroxylup-12-(13)-ene-17-carboxylic acid. This is a new compound reported for the first time in *S. alata* species. The spectral characterization of molecular structure of isolated compounds SA-1 and SA-4 was confirmed and supported using standard literature [17,18,19].

### 3.3. In Vitro COX-1 and COX-2 Inhibitory Assay

COX-1 and COX-2 catalyze the biosynthesis of prostaglandin H2 from the arachidonic acid substrate. The inhibition of COX-1 results in some undesirable side-effects, whereas COX-2 inhibition provides therapeutic effects in pain, inflammation, cancer, glaucoma, and Alzheimer’s and Parkinson disease [20]. COX-2 is an inducible enzyme, while COX-1 is constitutive, that is, present even in the absence of inflammatory conditions. In addition to the pro-inflammatory prostaglandins, COX-1 is responsible for the synthesis of those prostaglandins that are necessary for maintaining the integrity of gastro-intestinal mucosa. A higher inhibition of COX-1 increases the tendency of a drug to induce gastric ulcers and related complications [21].

The IC50 values of indomethacin for COX-1 and COX-2 were observed as 53.00 µM and 36.56 µM, respectively. The results of the in vitro COX-1 and COX-2 inhibitory studies revealed that the isolated compounds SA-1 and SA-4 potentially inhibit COX-2 (IC_50_ = 61.68–87.25 µM range) over the COX-1 104–128 µM range, whereas isolated compound SA-4 (COX-1/COX-2 = 104/61.68) were found to be potent inhibitor of COX-2 than SA-1 (COX-1/COX-2 = 128.4/87.25). To understand the inhibitory activity of these isolated compounds, they are further evaluated for their in vivo anti-inflammatory activity.

### 3.4. Anti-Inflammatory Activity

Anti-inflammatory activity of the phytocompounds (SA-1 and SA-4) was evaluated as per the method of Winter et al. with minor modifications. Carageenan was used to induced paw Edema in Wistar albino rat. Carrageenan induced paw edema is a standard assay for acute inflammation that is effectively employed to evaluate the phytoisolate against anti-inflammatory activity [22,23]. Edema is produced by a sequential release of inflammatory mediators such as histamine, serotonin, kinnins, prostaglandins and bradykinins, which leads to fluid accumulation [24]. Edema is characteristic of an acute inflammatory response [25]. The release of histamine or serotonin occurs in the first phase (up to 1 h) and the second phase (over 1 h) is associated with the production of bradykinins [26]. It is well known that the third phase of the edema induced by carrageenan, in which the edema reaches its highest volume, is characterized by the presence of prostaglandins and other compounds of slow reaction [27,28]. Previous studies have shown that phytocompounds of *S. alata* play an important pharmacological role in inflammation [29]. In the present study, among the two phytoisolates of *S. alata*, the compound SA-4 (24 mg/kg) was found to be more effective than SA-1 via the inhibition of the COX-1/COX-2 pathway. Results of the present study revealed that the phytoisolates SA-1 and SA-4 exhibit dose-dependent anti-inflammatory activity by suppressing the rat paw edema. The potent suppressive effect of SA-1 and SA-4 on inflammatory mediators was exerted by blocking of the expression of COX enzymes at different doses (2, 4, and 8 mg/kg). Additionally, macroscopical examination suggests that 24 mg/kg of *S. alata* can be an effective treatment for the management of inflammatory responses. These results suggest that phytoisolate of SA-4 could be a potent action for the anti-inflammatory effects. Moreover, several studies support that *S. alata* species have shown antioxidative effects [30,31]. Inhibition of COX by *S. alata* leads to a decrease in all prostaglandin and thromboxane synthesis, which accounts for the beneficial anti-inflammatory and prevents ulcerogenic effects [32]. Epidemiological evidence has convincingly demonstrated, through the inhibition of COX enzymes, the reduction in cancer risk [33]. Based on the antioxidant effect, SA4 may involve the inhibition of cyclooxygenase/prostaglandin-endoperoxide synthase (PGHS-1 and PGHS-2), and regulatory enzymes, involved in the biosynthesis of prostaglandin (PG) which is strongly implicated in inflammation [34]. 

### 3.5. Ulcerogenic Activity

It has been reported that non-steroidal anti-inflammatory agents are inadequately dissolvable in gastric acid and stay in contact with the stomach wall for a more extended period, thus producing a highly dangerous local concentration. This leads to local irritation of the stomach wall followed by ulceration. This prompts to local irritation of the stomach wall after ulceration [35]. Both the compounds SA-1 and SA-4 possessing in vivo anti-inflammatory activity were further screened for their ulcerogenic activity according to the Cioli method. The compounds SA-1 and SA-4 were administered to the animals via oral gavage (6 mg/kg, 12 mg/kg, and 24 mg/kg). Moreover, the results of the current study expressed that the action on inhibition of the ulcerogenic effect depends on different doses of SA-1 and SA-4 (6, 12, 24 mg/kg). SA-4 at the dose of 24 mg/kg showed significant ulcerogenic potential compared to the control treated group at doses of 6 and 12 mg/kg. Figure 3 presents the macroscopic view of effect of SA-1 and SA-4 on gastric mucosal lesions in ulcer model. The standard drug of indomethacin dose was chosen based on the standard references, which used Indoimethacin up to 40 mg [36,37]. As per Table 4, the anti-inflammatory activity of compounds SA-1 and SA-4 (administered at doses from 2 to 8 mg/kg) is comparable with Indomethacin (administered at the dose of 21 mg/kg, po). Table 5 expressed that ulcerogenic activity of compounds SA-1 and SA-4 (6–24 mg/kg) is also comparable to high dose indomethacin (21 mg/kg). Several anti-inflammatory and antioxidants studies wherein indomethacin have been used as a standard to compare the anti-inflammatory activity of phytoisolates [38,39,40]. The present study was designed based on the standard studies, where a similar experimental design was used [41,42,43,44].

The present study intended to explore the new phytochemical entities in the aerial part of *S. alata* species and investigate their anti-inflammatory potential. Fortunately, the present study explored and isolated a new compound SA-4 (3-hydroxylup-12-(13)-ene-17-carboxylic acid), which is reported for the first time in the *S. alata* species. Apart from that, this is the first time the anti-inflammatory potential of 3-hydroxylup-12-(13)-ene-17-carboxylic acid has been explored in *S. alata* species. So, this research acclaims its edge and novelty over other studies on the *S. alata* plant. A strong correlation between the potency of NSAID’s as an inhibitor of prostaglandin synthesis and ulcerogenic activity has been observed [45,46]. Most potent compound SA-4 showed a severity index lower than the standard drug Indomethacin. Hence, this compound may have better safety margin on gastric mucosa than indomethacin. 

## 4. Materials and Methods

### 4.1. General

The chemical and solvents used were purchased from commercial vendors and used without purification. The melting points of all isolates were determined in open capillary tube and were uncorrected. Ultraviolet spectra were recorded in methanol (MeOH) on a Shimadzu UV-160A UV visible recording spectrophotometer (Shimadzu Scientific Instruments, Kyoto, Japan). IR spectra were recorded on Bruker ATR-FTIR spectrophotometer. Mass spectra were recorded using electron impact ionization at 70 eV on an ESIMS analyst QTOF mass spectrometer (Agilent, Mississauga, ON, Canada). The ^1^H-NMR, ^13^C-NMR, and HMBC spectra were taken on a Bruker-Advance III-010601AM-500 spectrometer (Bruker Corporation, Karlsruhe, Germany) in CDCl_3_ and D_2_O using TMS as an internal standard expressing coupling constants (*J* values) in Hertz (Hz). Silica gel G (Qualigen, 60–120 mesh) was used for column chromatography. TLC was performed on plates coated with silica gel G (Merck, Darmstadt, Germany).

### 4.2. Plant Material and Extract Preparation

The plant material was supplied by Almas Pharmaceutical Ltd., Uttar Pradesh, India and identified by Dr. H.B Singh, Scientist F and Head, Raw Material Herbarium and Museum, NISCAIR (National Institute of Science Communication and Information Resources) Pusa Gate, New Delhi. The voucher specimen (NISCAIR/RHMD/2013/2185/190) of the test drug has been deposited in the herbarium of NISCAIR, India for future reference. The aerial parts were carefully collected and air dried under shade. The air-dried materials were reduced to coarse powder. The coarse powdered material (1.2 kg) was subjected to exhaustive extraction with 95% ethanol in a SOXHLET apparatus for 50 h. The extract was concentrated in rotary evaporator to yield greenish brown color 52.63 gms (4.385%) residue.

### 4.3. Isolation and Purification

The isolation of phytoisolates was based on the standard protocol with minor modification [47]. Briefly, the ethanolic extract (50 g) of *S. alata* was subjected to column chromatographic critical isolation by dissolving in the minimum volume of ethanol and adsorbed on the silica gel (60–120 mesh) slowly for preparation of a slurry. The extract was air-dried, powdered, and passed through a sieve (No. 8) to get uniform particle size. The clean and dried column plugged on the lower side with nonabsorbent cotton was fixed in a vertical position on the stand. After the column was half filled with petroleum ether, the silica gel (60–120 mesh) for column chromatography was poured in small portions and allowed to settle to form a stationary phase. The dried slurry of *S. alata* extract was loaded over the column and eluted successively with various combinations of chloroform: petroleum ether (1.5:8.5, 1.6:8.4, 1.7:8.3, 1.8:8.2, 1.9:8.1, 2:8, 2.1:7.9, 2.2:7.8, 2.3:7.7, 2.4:7.6, 2.5:7.5, 2.6:7.4, 2.7:7.3, 2.8:7.2, 2.9:7.1, and 3:7). The collected fractions homogeneity was checked by thin layer chromatography (TLC). Fractions with same retention factor (Rf) values were combined and concentrated. The concentrate was purified by preparative TLC using suitable solvent system to offer the pure phytoisolates SA-1 and SA-4. The purified phytoisolates were subjected to ultraviolet (UV), attenuated total reflection-Fourier-transform infrared (ATR-FTIR), nuclear magnetic resonance (1H-NMR, ^13^C-NMR and HMBC) and mass spectrometric studies for their structural elucidation. 

### 4.4. Animals

The animals (Wistar albino rats) used in the study were procured from Animal House Center and were divided and housed in different cages at 25 to 28 °C, under well maintained hygienic and environmental conditions with a relative humidity of 50 to 65%, under 12 h light and dark cycles. All animals were acclimatized for a week before use. All experimental work was conducted after receiving the approval from Institutional Animal Ethics Committee (IAEC) via protocol no. IAEC/2015-I/Prot no. 09, 10 and IAEC/2016-I/Prot no. 10, Delhi Institute of Pharmaceutical Sciences and Research, New Delhi. 

### 4.5. In Vitro Activity

The isolated compounds were screened for their in vitro COX-1 and COX-2 enzymatic activity using an enzyme immunoassay kit (catalog No. 560131, Cayman Chemicals Inc., Ann Arbor, MI, USA). The enzymatic assay was performed as per the manufacturer’s assay instructions and standard literature [48]. COX-1 and COX-2 enzymatic activity provides a simple, sensitive, and high-throughput adaptable method to detect the peroxidase activity of COX in biological samples or purified/crude enzyme preparations. The kit includes COX-1 and COX-2 specific inhibitors to differentiate the activity of COX-1 and COX-2 as well as other peroxidases, which may be present in the sample [49]. The efficiencies of the test compounds that cause 50% inhibition of COX-2 were calculated as IC50 from the concentration-response curve. The compounds were further tested for their in vivo anti-inflammatory activity. Our study showed the phytoisolate of SA-4 (COX-1/COX-2 inhibition activity of 104/61.68 µM with % inhibition of 61.36) to be more effective than SA-1 (COX-1/COX-2 inhibition activity of 128.4/87.25 µM, with % inhibition of 47.72).

### 4.6. In Vivo Activity

#### 4.6.1. Anti-Inflammatory Activity

The anti-inflammatory activity of isolated compound was evaluated on Wistar albino rat using carrageenan induced rat paw edema method as per the procedure described in earlier research [50]. The animals were divided into groups, consisting of five rats in each group. The prepared compounds were administered orally at doses of 2, 4, 8 mg/kg body weight (oral gavage) and the volume of paw was determined plethysmographically (Ugo-Basile, Gemonio (VA), Italy). The control group received the equivalent volume of normal saline and indomethacin (8 mg/kg b.wt.) was administered orally to the reference group. Carrageenan (0.1 mL, 1.0% *w*/*v* in 0.9% of normal saline) was injected after half an hour into the sub-plantar tissue of the rat’s hind paw. The paw volume was measured at hourly intervals for 3 h (0, 1, 2, and 3 h) and the percent inhibition of edema was calculated using the following formula:

% inhibition = (1 − V_s_/V_c_ × 100), (1)

where, V_s_ = paw volume in sample treated group, V_c_ = paw volume in control group.

#### 4.6.2. Acute Ulcerogenic Activity

The ulcerogenic activity of the isolated compound was performed according to previous reported method [51]. Each study group consisted of five Wistar albino rats. The animals were fasted for 18 h before the administration of the test compound, while water was given continuously. The dose quantity was made three times (6, 12, 24 mg/kg) of the administered dose for anti-inflammatory studies (2, 4, 8 mg/kg). The control group received only normal saline. After 6 h of drug administration the rats were sacrificed, the stomach was removed and opened around the greater curvature. The inner lining was washed properly with distilled water followed by normal saline. The mucosal damage was examined, and the number of ulcers and severity index was calculated on a scale of 0 to 3, where: 0 = no lesions; 0.5 = redness; 1.0 = spot ulcers; 1.5 = hemorrhagic streaks; 2.0 = ulcers > 3 but ≤5; 3.0 = ulcers > 5.

### 4.7. Statistical Analysis

Experimental data are expressed as mean. Statistical difference between the treated group and the control group was evaluated by one way analysis of variance (ANOVA) followed by Turkey’s test as a post ANOVA (Graph Pad Prism 5, San Diego, CA, USA) to determine the statistical significance. The results were considered statistically significant with *p* < 0.05.

## 5. Conclusions

The present study was intended to isolate and characterize the newer compounds i.e., SA-1 and SA-4 in *Swertia alata*. The isolated compounds were characterized and investigated for their anti-inflammatory and ulcerogenic properties. The findings of the current study concludes that among the two phytoisolates of *Swertia alata*, the compound SA-4 possess high anti-inflammatory potential and offers less ulcerogenic and less gastric irritant effect. The current study recommends that in the future histopathological examination and further metabolic studies should be done at a molecular level to support the molecular mechanism of action of phytoisolates of *Swertia alata* in the in vivo models.

## Figures and Tables

**Table 1:** ^1^HNMR and ^13^CNMR data for compound SA-1.

Position	^1^H NMR	^13^C NMR
1	1.18, m, 2H	29.26
2	1.79, m, 2H	29.61
3	3.56, m, 1H	29.38
4	--	77.02
5	1.18, m, 1H	29.26
6	1.42, m, 2H	29.38
7	1.18, t, 2H (*J* = 7.22)	29.26
8	--	33.75
9	2.13, t, 1H (*J* = 7.19)	35.24
10	--	29.61
11	2.30, d, 2H (*J* = 7.19)	35.32
12	5.21, t, 1H (*J* = 15.12)	125.77
13	--	127.28
14	--	35.46
15	1.89, t, 2H (*J* = 7.26)	29.61
16	2.5, t, 2H (*J* = 7.26)	24.71
17	--	35.52
18	2.27, t, 1H (*J* = 7.12)	35.62
19	1.18, t, 2H (*J* = 7.12)	29.26
20	--	31.94
21	1.18, m, 2H	29.26
22	2.13, t, 2H (*J* = 7.25)	23.76
23	0.4, s, 3H	14.1
24	0.45, s, 3H	14.1
25	0.52, s, 3H	22.71
26	0.78, s, 3H	29.06
27	0.81, s, 3H	29.06
28	--	178.33
29	0.47, s, 3H	24.71
30	0.47, s, 3H	24.71

Note: Coupling constants in Hertz are provided in parenthesis.

**Table 2:** ^1^HNMR and ^13^CNMR data of compound SA-4.

Position	^1^H NMR	^13^CNMR
1	1.18, m, 2H	27.2
2	1.70, m, 2H	27.96
3	3.72, m, 1H	72.53
4	--	27.82
5	1.18, m, 1H	27.2
6	1.45, m, 2H	27.82
7	1.18, m, 2H	27.2
8	--	33.25
9	1.18, m, 1H	35.28
10	--	27.96
11	2.13, t, 2H (*J* = 7.6)	35.32
12	5.20, t, 1H (*J* = 15.1)	125.42
13	--	127.35
14	--	35.46
15	1.18, m, 2H	27.96
16	1.70, m, 2H	24.97
17	--	35.52
18	2.23, d, 1H (*J* = 7.2)	35.62
19	1.56, m, 1H	51.63
20	1.90, d 2H, (*J* = 12.2)	35.62
21	1.96, d, 2H, (*J* = 12.2)	35.92
22	0.81, s, 3H	17.97
23	0.85, s, 3H	17.97
24	0.95, m, 3H	24.97
25	0.95, m, 3H	18.01
26	0.95, m, 3H	27.2
27	--	27.2
28	1.56, m, 1H	
29	0.65, d, 3H (*J* = 7.22)	17.97
30	0.65, d, 3H (*J* = 7.22)	17.97

Note: Coupling constants in Hertz are provided in parenthesis.

**Table 3:** IC_50_ of the synthesized compounds by in vitro COX-1 and COX-2 enzymatic assay and COX-2.

Position	IC_50_ (µM)	
	COX-1	COX-2
SA-1	128.4	87.25
SA-4	104	61.68
Indomethacin	53	36.56

Note: C_50_ value is the concentration of the compound required to produce 50% of inhibition of COX-1 and COX-2 respectively using enzyme immuno-assay kit (Catalog No. 560131, Cayman Chemicals, Inc., Ann Arbor, MI, USA).

**Table 4:** In vivo anti-inflammatory activity of compounds using carrageenan-induced rat paw edema method.

Compound		Increase in Paw Edema (mL) ^a,b^ (Mean ± SEM)
SA-1	2 mg/kg	0.29 ± 0.00
	4 mg/kg	0.27 ± 0.22
	8 mg/kg	0.23 ± 0.01
SA-4	2 mg/kg	0.28 ± 0.00
	4 mg/kg	0.26 ± 0.22
	8 mg/kg	0.17 ± 0.01
Control		0.44 ± 0.04
Indomethacin		0.15 ± 0.02

Note: *p*-values were compared with the control group (3 h after inducing edema) (Tukey’s test). Number of animals (rats) in each group = 5. ^a^ Values are determined after 3 h and are expressed as Mean ± SEM. ^b^ *p* < 0.05 (significant difference).

**Table 5:** In vivo ulcerogenic activity of the compounds in rat model.

Compound		Ulcerogenic Activity ^a^ (Severity Index) ^b,c^ (Mean ± SD)
SA-1	6 mg/kg	0.0 ± 0.00
	12 mg/kg	0.0 ± 0.00
	24 mg/kg	2.5 ± 0.61
SA-4	6 mg/kg	0.0 ± 0.00
	12 mg/kg	1.8 ± 0.44
	24 mg/kg	2.1 ± 0.44
Control		0.0 ± 0.00
Indomethacin		2.7 ± 0.27

Note: ^a^ Number of animals in each group is 5. ^b^ Severity Index = mean score of treated group and mean score of control group. ^c^ *p* < 0.5 (significant difference).

## Data Availability

The data presented in this study are available in the article.
